# Rehabilitation needs of adults after a brain tumour diagnosis: A scoping review

**DOI:** 10.1371/journal.pone.0325266

**Published:** 2025-07-17

**Authors:** Bernadine O’Donovan, Niamh Kavanagh, Ailish Malone, Frances Horgan, Kathleen Bennett

**Affiliations:** 1 School of Population Health, RCSI University of Medicine and Health Sciences, Dublin, Ireland; 2 Neuro-Oncology, Beaumont Hospital, RCSI Smurfit Building, Dublin, Ireland; 3 School of Physiotherapy, RCSI University of Medicine and Health Sciences, Dublin, Ireland; IRCCS Medea: Istituto di Ricovero e Cura a Carattere Scientifico Eugenio Medea, ITALY

## Abstract

Rehabilitation can improve physical and cognitive function and quality of life in people who have been diagnosed with brain tumours. The aim of this scoping review was to examine the international evidence related to the rehabilitation needs and interventions of people diagnosed with a brain tumour. Relevant search terms were used to identify eligible studies and five databases were searched for original work published between January 2003 and December 2023. A total of 48 studies were included in the final review. A wide range of unmet rehabilitation needs across physical, cognitive, emotional or social domains were reported. In addition, a variety of interventions to improve function after a brain tumour diagnosis, have been investigated across healthcare settings. Our results indicated that rehabilitation needs can be prolonged and evolve over time which has implications for survivorship care. Many intervention studies investigating cognitive and physical rehabilitation reported beneficial outcomes. However, the relevant evidence is not implemented in current practice nor has widely contributed to the development of clinical practice guidelines to inform rehabilitation approaches for those with a brain tumour. This scoping review suggests limited evidence on patient-level needs, such as returning to work or community and social networks, and future research in these areas is recommended. Research which focuses on optimising interventions and the role of multidisciplinary teams is also required.

## Background

Brain and central nervous system (CNS) cancer contributes to the global burden of disease, estimated 19th among the most common malignancies and 12th among the leading causes of cancer deaths worldwide [1]. Brain tumours can shorten lifespan and neurological impairments can create significant symptom burden [2]. Brain tumour survivors can experience poor quality of life, with symptoms leading to increased depression, anxiety or apathy [3]. There may also be financial strain as an individual’s ability to work and travel may be limited [3]. Rehabilitation is a process that seeks to maximise patients’ quality of life and independence. The core values of rehabilitation practice include holistic collaboration among multi-disciplinary teams, goal setting, education and training for patients and their families. Early rehabilitation is important to improve patients’ functioning in daily life and reduce potential complications [4]. However, rehabilitation after a brain tumour diagnosis is complicated by long treatment regimens and numerous side effects, which may make determining the optimal timing of rehabilitation interventions difficult [5].

Furthermore, physical and cognitive impairments can fluctuate during and after treatment. These changes have implications for goal-setting in rehabilitation practice. Specifically, the type of interventions offered to patients, restorative or adaptive, will be impacted by the type of treatment they receive. Despite a general consensus on the therapeutic value of rehabilitation there is limited information on the rehabilitation needs and use of neuro-rehabilitation services by people who have been diagnosed with a brain tumour [6]. However, some evidence has emerged that rehabilitation can improve quality of life and functional outcomes (motor and cognitive) in people after brain tumour diagnosis [7–9]. More widely, research in acquired brain injury has shown that early access to rehabilitation can improve patient physical and cognitive outcomes and reduce complications and time in hospital [4,10]. It is recognised that people who have been diagnosed with a brain tumour often experience difficulties accessing rehabilitation services; with lack of adequate provision a feature of existing neuro-rehabilitation services [4]. Considering the potential benefits of rehabilitation, there is a need to examine current evidence on rehabilitation needs and interventions to support rehabilitation in those with a brain tumour in order to guide effective service provision.

### Aims/objectives

The aim of this scoping review was to examine international evidence on rehabilitation needs of adults who have been diagnosed with a brain tumour and describe types of rehabilitation interventions post-diagnosis and/or treatment. The specific objectives of the review are:

1) To determine the extent and range of current evidence on rehabilitation needs in adults after a brain tumour diagnosis.
2) To describe the type and range of interventions (physical and cognitive) for adults after a brain tumour diagnosis and/or treatment.

### Methods

The overall conduct of the scoping review was informed by the Joanna Briggs Institute (JBI) framework, while the Preferred Reporting Items for Systematic Reviews and Meta-analysis extension for scoping reviews (PRISMA-ScR) was also followed [11]. See Table in S1 Table. A protocol for this review was previously published [12].

### Search strategy

The search strategy was determined by the research team in consultation with a specialist librarian. Combinations of disease terms, rehabilitation terms and terms related to rehabilitation needs were adapted from previous research [8]. The following electronic databases were searched: Embase, CINAHL Complete, Medline (Pubmed), PsycINFO and Physiotherapy Evidence Database PEDro; from January 2003– December 2023 inclusive and restricted to English language papers only. The full search terms are available in Table in S2 Table.

### Eligibility criteria

Rehabilitation needs were defined as any difficulties that might reduce functioning/cause disability, measured with quantitative/qualitative methods. The International Classification of Functioning, Disability and Health (ICF) framework was used to identify needs pertaining to motor, sensory, cognitive, speech functions and activities of daily living, including moving around, transferring oneself or walking [13]. Inclusion criteria were: (i) studies of adult patients (≥18 years); (ii) study participants had a confirmed diagnosis of brain tumour according to WHO Classification of Central Nervous System tumours (WHO, 2021, or earlier version) (iii) studies that examine the rehabilitation needs of people with primary/secondary (metastatic) brain tumours and (iv) studies that describe patient outcomes related to rehabilitation (physical and cognitive). Opinion pieces, editorials and commentaries were excluded. Reviews were checked to identify any potentially eligible articles that might have been missed by the electronic searches. Consistent with the purpose of scoping reviews to give a broad overview of existing research, quality assessment of the selected literature was not reported. Full details of inclusion/exclusion criteria are presented in Table in S3 Table.

### Data extraction

At least two reviewers independently screened titles and abstracts of records with Covidence software (KB, FH, AM, BO’D). Full text versions of potentially eligible papers were examined to assess their suitability. Any disagreements were resolved by consensus. Data was extracted for the following fields: (a) authors, study design, year and country of study; (b) study population; (c) rehabilitation need investigated/rehabilitation intervention (as per WHO package of interventions for rehabilitation: module 7: malignant neoplasms) [14] (d) data collection method and instruments used, and (e) main results. A narrative synthesis was undertaken based on (i) overall rehabilitation needs of adult patients who have been diagnosed with brain tumour in Ireland and (ii) evidence of type/range of interventions.

### Framework for evidence synthesis

The WHO Package of interventions for rehabilitation is a resource for planning for the integration of rehabilitation services into countries’ health systems. It summarises essential interventions for rehabilitation for 20 health conditions including malignant neoplasms. Information on delivery of these interventions – required assistive products, equipment, consumables and workforce – is linked to each intervention [14]. The WHO Package of interventions of rehabilitation: module 7 was developed for use in the rehabilitation of children and adults with malignant neoplasms of any location or specification. The interventions focus on fourteen elements of functioning that are relevant to malignant neoplasms: 1.mental/cognitive functions, 2.pain management, 3.bowel and bladder management, 4.sexual function and intimate relationships, 5.cardiovascular and immunological functions, 6.motor functions and mobility, 7.exercise and fitness, 8.activities of daily living (ADL), 9.interpersonal interactions & relationships, 10.education and vocation, 11.community and social life, 12.lifestyle modification, 13.self-management and 14.carer and family support. See Table 1 for summary of WHO functions and rehabilitation needs/targets.

**Table 1:** Summary of WHO functions and rehabilitation needs/targets.

WHO functions 1–14	Rehabilitation needs/targets
Mental/cognitive functions	Fatigue Sleep functions Body image Cognitive functions
Pain management	Sensation of pain
Bowel and bladder management	Urination & defecation functions
Sexual functions & intimate relationships	Sexual functions & intimate relationships
Cardiovascular and immunological functions	Oedema control Vasomotor symptoms (hot flashes, night sweating)
Motor functions and mobility	Joint mobility Muscle power functions Involuntary movement reaction functions Gait pattern functions Mobility
Exercise and fitness	Exercise tolerance functions
Activities of daily living	Activities of daily living (ADL)
Interpersonal interactions & relationships	Interpersonal interactions &relationships
Education and vocation	Education Work & employment
Community & social life	Participation in community & social life
Lifestyle modification	Healthy lifestyle
Self-management	Self-management
Carer & family support	Carer & family support

## Results

**Fig 1:**
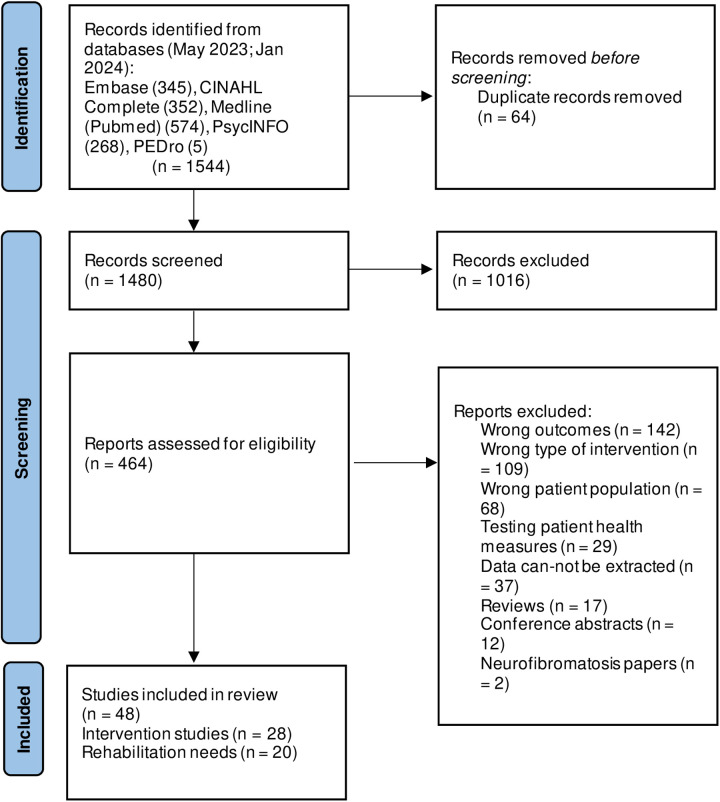
PRISMA flowchart for the scoping review of rehabilitation needs in adults with a diagnosis of a brain tumour.

A total of 1,544 records were identified from databases and hand searches. These were reduced to 1,480 following removal of duplicates. During title and abstract screening 464 articles were selected for full text review. This resulted in 48 papers that met the eligibility criteria and were included in the scoping review (47 studies). Figure 1 shows the number of papers identified, screened and included.

### Characteristics of studies

A total of 48 papers reporting 47 studies were included: 20 papers related to rehabilitation needs with 15 quantitative [15,17–21,23–29,31,32,34]; three mixed methods [16,22,30] and one qualitative study [33] and 28 intervention studies [35–62]. A variety of study designs were reported, specifically, randomized controlled trials (n = 13), prospective/retrospective cohort studies (n = 19) and eight feasibility studies. The research was conducted from 2003–2023 in 15 different countries: USA (n = 15); Australia (n = 6); Denmark (n = 5); Italy (n = 4); The Netherlands (n = 4); Canada (n = 3); Germany (n = 2); Poland (n = 2); Korea (n = 2); Malaysia (n = 1); France (n = 1); Japan (n = 1); Colombia (n = 1); India (n = 1) and UK (n = 1). Sample sizes varied across studies ranging from four to 1,722 participants. Most of the studies did not report gender – there was one paper with a male only population [40]. A variety of tumour types were investigated – for information on tumour classifications see Figure in S1 Figure. Many studies (n=22; 46%) focused on high grade tumour diagnoses [17,21,26, 30, 34,–37, 40–43, 45, 46, 49–53, 55, 57, 58, 62] with sample sizes ranging from 4-131. Four papers with 15-140 participants reported on low grade tumours [16, 33, 42, 60] and 2 studies [28,31] included both high and low grade tumours (n = 38 and n = 116 respectively).  

For the purposes of data extraction studies were divided in two groups: studies related to rehabilitation needs and intervention studies. The main findings from all the studies are presented in Table in S4 and table in S5 Table. Demographic and study characteristics are summarised in Tables 2 and 3.

**Table 2:** Summary and characteristics of studies related to rehabilitation needs (n = 20).

Authors [reference]	Number of participants	Type of tumour	Need investigated	Rehabilitation needs/targets (WHO functions 1–14)^*^
Acquaye et al., 2017 [15]	114	Brain ependymoma	Physical – symptom burden Social – work Activities of daily living (ADL)	Mental/ cognitive functions (1), Pain management (2), Motor functions and mobility (6), Activities of daily living (8), Education and vocation (10)
Affronti et al., 2018 [16]	15	Low-grade glioma	Physical – symptom burden Coping strategies QoL	Mental/ cognitive functions (1), Self-management (13)
Aprile et al., 2015 [17]	67	High grade glioma (HGG)	Physical – fatigue, function Psychological – distress QoL	Mental/ cognitive functions (1), Motor functions and mobility (6)
Benz et al., 2018 [18]	1722	Intracranial meningioma	QoL	Mental/ cognitive functions (1), Motor functions and mobility (6), Community & social life (11)
Boele et al., 2015 [19]	65	Stable LGG	HRQoL	Mental/ cognitive functions (1)
[20] Cantisano et al., 2021 [20]	80	Primary brain tumour	Executive functions (EFs)	Mental/ cognitive functions (1)
Halkett et al., 2022 [21]	116	HGG	Supportive care needs Emotional –distress Physical -wellbeing over time	Mental/ cognitive functions (1)
Kearney et al., 2022 [22]	37	Left-hemisphere tumours	Cognitive & Emotional – Language HRQoL	Mental/ cognitive functions (1)
Khan et al., 2013 [23]	106	WHO central nervous system tumours	Physical – pain, motor function, cognitive function QoL Survivor care needs	Mental/ Cognitive functions (1), Pain Management (2), Motor functions and mobility (6)
Kim et al., 2012 [24]	25	Brain tumours (resected)	Fatigue Motor function Mood QoL	Mental/ cognitive functions (1), Motor functions and mobility (6)
Krajewski et al., 2023 [25]	92	Primary brain tumours (malignant & non malignant)	Functional status Gait efficiency ADL LoS	Motor functions and mobility (6), Activities of daily living (8)
Kvale et al., 2009 [26]	50	GBM	Distress QoL	Mental/ cognitive functions (1)
Lowe et al., 2014 [27]	31	Brain metastases	Physical activity (PA) Symptoms QoL	Mental/ cognitive functions (1), Motor functions and mobility (6)
Miklja et al; 2022 [28]	38	Low- or high-grade glioma	Physical activity Exercise HRQoL	Mental/ cognitive functions (1), Exercise and fitness (7)
Pace et al., 2016 [29]	719	Primary brain tumour	Pattern of rehab care 12 months after diagnosis Motor rehab Language/cognitive rehab	Mental/ cognitive functions (1), Motor functions and mobility (6)
Piil et al., 2017 [30]	63 (30 pts & 33 caregivers)	Newly diagnosed with HGG	Physical activities QoL Anxiety Depression	Mental/ cognitive functions (1), Motor functions and mobility (6)
Porensky et al., 2013 [31]	116	Brain tumour high-grade & low-grade	QoL Distress Symptoms – cognitive & physical	Mental/ cognitive functions (1), Motor functions and mobility (6)
Reinert et al., 2020 [32]	44 Pts & caregivers	Brain tumour	Psycho-oncologic need Depression Information need	Mental/ cognitive functions (1), Self-management (13), Carer & family support (14)
Rimmer et al., 2023 [33]	28	Lower-grade gliomas (LGG 13	Self-management	Self-management (13)
Umezaki et al., 2020 [34]	76	WHO grade II-IV glioma	Symptom burden QoL	Mental/ cognitive functions (1), Motor functions and mobility (6), Activities of daily living (8)

* WHO package of interventions for rehabilitation: module 7: malignant neoplasms categories 1–14; GBM = Glioblastoma; pts = patients.

**Table 3:** Summary and characteristics of intervention studies (n = 28).

Authors [reference]	Number of participants	Type of tumour	Rehabilitation intervention	Rehabilitation needs/targets (WHO functions 1–14)^*^
Baima et al., 2017 [35]	15	High-grade brain tumours	Novel strength & balance home exercise program	Exercise and fitness (7)
Boele et al., 2018 [36]	115	Glioma patients	Online guided self-help intervention for managing depression	Mental/ cognitive functions (1), Self-management (13)
Clarke et al., 2013 [37]	131	Patients with advanced cancer	Multidisciplinary intervention (RCT) to improve QOL	Mental/ Cognitive functions (1), Pain Management (2), Interpersonal interactions & Relationships (9), Community & social life (11), Lifestyle Modification (12), Self-management (13), Carer & family support (14)
Culos-Reed et al., 2017 [38]	16	HGG brain cancer patients	Feasibility of an exercise study	Motor functions and mobility (6), Exercise and fitness (7)
Dahlberg et al., 2022 [39]	19	Persons living with brain tumours	Testing of social network-mapping tool	Interpersonal interactions & relationships (9), Community & social life (11), Self-management (13)
Fahrenholtz et al., 2019 [40]	5	Primary glioma	Physical therapy and occupational therapy	Mental/ cognitive functions (1), Motor functions and mobility (6), Activities of daily living (8), Self-management (13)
Fouda et al., 2023 [41]	4	WHO grade I intracranial meningioma	Evaluation of Cogmed Working Memory Training (CWMT) program	Mental/ cognitive functions (1)
Gehring et al., 2009 [42]	140	Low-grade anaplastic gliomas	Evaluation of cognitive rehabilitation program (CRP)	Mental/ cognitive functions (1)
Gehring et al., 2020 [43]	34	WHO grades II/III glioma	Exercise intervention (RCT)	Mental/ cognitive functions (1), Exercise and fitness (7)
Sandler et al., 2020 [44]	12	Primary brain cancer	Individualised exercise intervention	Mental/ cognitive functions (1), Motor functions and mobility (6), Exercise and fitness (7)
Hansen et al., 2018 [45]	24	Patients with glioma	Interdisciplinary rehabilitation intervention	Motor functions and mobility (6), Exercise and fitness (7)
Hansen et al., 2020 [46]	64	Patients with gliomas	Supervised rehabilitation physical therapy & occupational therapy	Mental/ cognitive functions (1), Motor functions and mobility (6), Exercise and fitness (7), Activities of daily living (8)
Maialetti et al., 2020 [47]	33	Brain tumuor	Multimodal rehabilitation pathway (MRP)	Mental/ Cognitive functions (1), Community& social life (11)
McCarty et al., 2017 [48]	49	Malignant brain tumours	Interdisciplinary outpatient rehabilitation program	Mental/ cognitive functions (1), Motor functions and mobility (6), Activities of daily living (8), Education and vocation (10)
Milbury et al., 2018 [49]	10 (5 pts, 5 caregivers)	HGG	Dyadic yoga program (DYP)	Mental/ cognitive functions (1), Exercise and fitness (7), Carer & family support (14)
Nordentoft et al., 2022 [50]	33 (17 pts, 16 carers)	High-grade glioma	Evaluation of REHPA-HGG multimodal rehabilitative palliative care program	Mental/ cognitive functions (1), Motor functions and mobility (6), Education and vocation (10), Carer & family support (14)
Ooi et al., 2013 [51]	38	Primary intracranial tumours	Rehabilitation interventions	Mental/ cognitive functions (1), Motor functions and mobility (6)
Ownsworth et al., 2015 [52]	50	Primary brain tumour	Home-based psychosocial intervention Making Sense of Brain Tumour (MSoBT) program (RCT)	Mental/ cognitive functions (1), Self-management (13)
Ownsworth et al., 2023 [53]	118 - 82 pts & 36 caregivers	Primary brain tumour	Remote delivery of MAST (Tele‐MAST) via telephone & videoconferencing (Making Sense of Brain Tumour)	Mental/ cognitive functions (1), Community & social life (11), Self-management (13)
Pace et al., 2007 [54]	121	Malignant brain tumour	Post-discharge rehab home care included neuro rehabilitation	Mental/ cognitive functions (1), Motor functions and mobility (6)
Pieczyńska et al., 2023 [55]	47	HGG	Augmented reality-based rehabilitation exercises remote program	Mental/ cognitive functions (1), Motor functions and mobility (6), Exercise and fitness (7)
Rhudy et al., 2023 [56]	16 8 pts & 8 caregivers	Acquired brain disorders	Resilient Living program, psychosocial intervention	Mental/ cognitive functions (1), Motor functions and mobility (6), Self-management (13), Carer & family support (14)
Richard et al., 2019 [57]	25	Primary brain tumour	Evaluation of Goal Management Training (GMT) (RCT)	Mental/ cognitive functions (1), Activities of daily living (8), Self-management (13)
Spencer et al., 2021 [58]	30	High-grade glioma	Evaluation of 10-week exercise intervention	Mental/ cognitive functions (1), Motor functions and mobility (6)
Troschel et al., 2020 [59]	15	Brain tumours	Evaluation of ski exercise intervention	Mental/ cognitive functions (1), Exercise and fitness (7)
Van der Linden et al., 2021 [60]	62	Low-grade glioma & meningioma	Evaluation of tablet-based cognitive rehabilitation program (ReMind)	Mental/ cognitive functions (1), Self-management (13)
Yoon et al., 2015 [61]	40	Brain tumour	Evaluation of virtual reality-based rehabilitation (RCT)	Motor functions and mobility (1), Activities of daily living (8)
Zucchella et al., 2013 [62]	58	Primary brain tumours	Evaluation of early cognitive rehabilitation	Mental/ cognitive functions (1)

* WHO package of interventions for rehabilitation: module 7: malignant neoplasms); RCT = randomised control trial; pts = patients.

### Studies related to rehabilitation needs

Twenty studies were identified that reported on rehabilitation needs post-diagnosis, across a range of domains including physical, cognitive emotional, social, or information. See Table S4 Table. Several studies assessed both physical and emotional/cognitive needs, and each area is discussed separately. All of these studies included a mix of low-grade to high grade tumours and the study outcomes were categorised according to WHO functioning and rehabilitation targets [13,14]. Research focused on the following areas: physical needs (n = 13 studies; 65%); quality of life (n = 13 studies; 65%); psychosocial/ emotional (n = 10 studies; 50%); cognitive (n = 8 studies; 40%) and information needs (n = 2 studies; 10%). Rehabilitation needs were not ranked in importance by participants in these studies. Further information in Table in S5 Table.

### Physical needs

A total of 13 studies focused on physical needs post brain tumour diagnosis/tumour resection [15–17,21,23–25,27–31,34]. The focus of each of these studies was mainly physical symptom burden/wellbeing post diagnosis [15–17,21,27,28,31,34], motor function [17,23–25,29] and associated pain, gait efficiency and physical activity/exercise [37,38,42,45,46,49,55,58] (see Table in S6 Table).

A mixed methods study in the USA assessed individuals’ needs at specific intervals post low grade glioma diagnosis [16]. A total of 15 participants at a Southern Academic Brain Tumour Center completed qualitative interviews and quantitative assessments. The results demonstrated fatigue as the main physical symptom burden, with 40% experiencing this throughout the 6-month assessment post diagnosis.

Fatigue was also the focus of a cross-sectional study in Italy among 67 participants with a diagnosis of high-grade gliomas recruited from outpatient departments [17]. Sixteen patients were diagnosed with a high-grade anaplastic astrocytoma and 51 patients had a diagnosis of a glioblastoma multiforme. Through their analysis the study found that more than a third of patients had clinically relevant fatigue (Brief Fatigue Inventory ≥3) which impacted their daily life.

A retrospective cohort study in Seoul, Korea focused solely on the fatigue levels of patients diagnosed with primary brain tumour. Twenty-five participants who were in an inpatient rehabilitation facility recovering from brain tumour resection completed fatigue focused questionnaires on admission and after 4 weeks. The results of this study found that patients with a recurrent brain tumour experience more fatigue and that those who scored with moderate fatigue on admission to the rehabilitation unit had improved post rehabilitation [24].

A large prospective cross-sectional study in Australia, included 106 people with low grade and high grade brain tumours who were outpatients attending rehabilitation clinics across Melbourne [23]. The participants completed quantitative assessments which looked at both physical and cognitive needs such as communication issues, ataxia, limb weaknesses and cognitive changes. A highlight from the study is the psychosocial impacts of cognitive and social issues, as participants and family members noticed increased withdrawal from routine social gatherings. Results on a final 6-month assessment check in with participants indicated that rehabilitation improved some symptoms such as physical limb strength. In addition, participation in a social event such as rehabilitation appointments could also have benefits in daily life [23].

Overall studies described a range of physical issues such as fatigue, pain, mobility and muscle function which could have implications for daily activites – work/education – and relationships.

### Cognitive and emotional needs

In all 20 studies examined, there was a focus on cognitive and emotional needs of people with a diagnosis of a brain tumour [15–34]. The main findings of these studies found that memory/concentration, emotional problems including depression, nervousness, sadness, loss of interest in usual activities, general wellbeing, overall decrease in quality of life, and insomnia were mainly reported as impacting the lives of people after a diagnosis of a brain tumour. The main data collection tools used across all studies are the Karnofsky Performance Scale [17,24,25,30,34], the Functional Assessment of Cancer Therapy –Brain/Cognitive [16,22,26], the Beck Depression Inventory [16,24] and Brief Inventory Fatigue [17,24].

A Canadian cross-sectional study with 31 people with terminal cancer with brain metastases and receiving palliative whole brain radiotherapy was carried out in the Cross Cancer Institute. It focused on cancer related fatigue and the impact of physical activity [27]. A survey and activity tracker device were used to assess fatigue symptoms and quality of life (QoL). Their findings show higher rates of depression, anxiety and feeling of well-being in people who had lower physical activity rates than those who were more active [27].

However, another cross-sectional study in the USA found no correlation between higher levels of physical activity and lower levels of anxiety/depression/fatigue [28]. Thirty eight people diagnosed with low or high grade glioma and being treated at the University of Michigan Hospital, completed physical activity and health-related quality of life telephone surveys. Participants varied in whether the assessment was completed prior to commencement or during treatment.

An unmet need for counselling for both patients and their family caregivers to aid with depression and high psycho-oncologic need was highlighted in one study carried out in Germany [32]. This cross-sectional study was with 172 adult patients, with grade I-IV brain tumours treated in the Regensburg Brain Tumour Centre and 142 family caregivers. Participants received psycho-onocology care which had a positive impact on self-reported feelings of depression. This study was carried out when patients were undergoing first-line or recurrence treatment and used self-reported questionnaires.

A cross-sectional study with 76 patients with grade II–IV glioma and either in treatment or in follow-up observation was carried out in Tokyo. It highlighted the impact of health complications on daily life, such as financial implications, insomnia, appetite loss, drowsiness and uncertainty for the future [34].

Overall, the studies reported a range of rehabilitation targets/needs that included healthy lifestyle, self-management, participation in community/social life, activities of daily living, exercise tolerance, reactions, sensation of pain, and body image/sleep and cognitive functions.

### Intervention studies – physical therapy

Overall 28 intervention studies (physical and cognitive) for adults after a brain tumour diagnosis and/or treatment were identified. Sixteen studies involved physical rehabilitation [35,38,40,43–46,48–51,54–56,58,59,61]. - Ten intervention studies targeted the WHO function ‘Exercise and fitness’: home based exercise programs [35,38,43], individualised moderate-intensity, mixed-mode (aerobic and resistance) exercise intervention [44] , outpatient therapy (with supervised and non-supervised training) [45], supervised physical and occupational therapies [46], yoga program [49], augmented reality interventions [55,61], exercise classes [58] which included three exercise feasibility studies [38,45,58]. See Table in S5 Table and Table in S7 Table.

The majority of interventions were for patients, however, two studies included interventions for patients and caregivers/relatives – a dyadic yoga program [49] and a ski exercise program [60]. Most of the interventions took place in outpatient settings [35,38,43–46,58,59]. See Table in S7 Table.

Studies of outpatient rehabilitation reported beneficial outcomes with improved physical well-being and quality of life in most of the studies [35,43,44,46,49,58,59]. A randomised controlled trial (RCT) in the Netherlands enrolled 34 patients with WHO grades II/III glioma for home-based exercise therapy over six months. It involved three aerobic exercise sessions of moderate to vigorous intensity per week (20–45 minutes). The control group received motivational brochures with advice on active lifestyle in the first week and at 3 months. Participants in the exercise group displayed improvements in attention, information processing speed, verbal memory and executive function. There were also positive outcomes for this group in cognitive symptoms, fatigue, sleep, mood and health related quality of life (HRQoL) [43]. There were mixed findings in augmented reality interventions. A previous intervention study in South Korea of 40 patients diagnosed with brain tumour with upper-extremity (UE) dysfunction, used augmented reality rehabilitation (Neuroforma neurorehabilitation program). The intervention group engaged in a 30-minute virtual reality program for 9 sessions and 30-minute occupational therapy for 6 sessions over 3 weeks. The control group received 30-minute occupational therapy alone for 15 sessions over 3 weeks. It found significant improvements in physical function in each group [61]. However, a recent randomised clinical trial including 47 Polish patients with high grade glioma (HGG) which used augmented reality-based exercises (Interactive Rehabilitation and Exercise (IREX) System) reported no significant changes in physical/cognitive function, fatigue, mood or quality of life (QoL) in the exercise group [55]. Ten studies focused on the WHO function ‘Motor functions and mobility’ [44–46,48,50,51,54–56,61] with five describing multimodal rehabilitation programs [45,46,48,50,54]. A large Italian study of post-discharge neuro rehabilitation (home care) was conducted with 121 patients with brain tumour. Significant improvements in function and HRQoL were found after 3 months of rehabilitation [54]. A prospective, longitudinal US study with 49 patients with brain tumours found that patients who engaged with two/three multimodal therapies, at six day rehabilitation sites, for 35 months had higher physical functioning scores reported greater HRQoL and less pain [48].

### Intervention studies – cognitive therapy

Twenty-three intervention studies involved cognitive rehabilitation and focused on Mental/Cognitive functions [36,37,40–44,46–60,62]. Seven studies described psychosocial programmes and many also targeted the following WHO functions: ‘Self-management’ [36,37,39,40,52,53,56,57,60], ‘Activities of daily living [40,46,48,57,61], ‘Community & social life’ [37,39,47,53], ‘Carer & family support’ [37,49,50,56] ‘Education and vocation’ [48,50] ‘Interpersonal interactions & relationships’ [37,39], ‘Pain management’ [37] and ‘Lifestyle modification’ [37]. See table in S7 Table.

Nine interventions were available remotely with guidance from therapists [36,37,41–43,52,53,60,62]. The majority of interventions were for patients however, four studies had cognitive programs for patients and caregivers [37,49,53,56] with a dyadic yoga program leading to improved QoL in caregivers [49]. Many cognitive interventions involved multiple therapies [47,48,50,54,57,60] with a range of approaches such as neurocognitive training, skills training, supportive meeting groups [39,41,42,47,50,53,57,60,62]. Tailored cognitive programs were described in eight studies: social CareMaps [39], Cogmed Working Memory Training (CWMT) program [41], the multimodal rehabilitative palliative care program (REHPA-HGG) [29], Making Sense of Brain Tumor (MSoBT) [52], MAST (Tele‐MAST) (Making Sense of Brain Tumor) [53], Resilient Living program [56], Goal Management Training (GMT) [57], and tablet-based program ReMind [60]. Most of the interventions took place with patients who were post treatment/surgery in outpatient settings [36,37,40–44,46–49,51–55,57–60,62] -and two in acute inpatient rehabilitation [50,56]. Cognitive rehabilitation was associated with positive outcomes: improved memory, attention, executive/cognitive function [37,41–43,46,47,57] and increased QoL [36,37,44,46,49,51–54,58,62]. Interventions also resulted in reductions in mental fatigue [36,42–44,46,51,58], depression [44,52,53], cognitive related distress [47,59] and sleep disturbances [43,49]. A randomised controlled trial including 64 Danish glioma patients examined supervised rehabilitation – 6 weeks of physical therapy with an emphasis on cardiovascular/resistance training and individually tailored 60-minute occupational therapy for 2 sessions per week – and usual rehabilitation care. It found that supervised PT and OT therapies resulted in improved cognitive function, QoL and reduced fatigue [46]. A larger randomised control trial in Australia conducted the Tele-MAST intervention. Ten cognitive rehabilitation sessions per week, of one hour duration – were administered via Zoom videoconferencing with 118 patients with primary brain tumour and caregivers [53]. Ten cognitive rehabilitation sessions per week, of one hour duration, were delivered via Zoom videoconferencing. The intervention group received psychoeducation, psychotherapy and strategy training to address cognitive effects. Improvements in global QoL, emotional QoL and lower anxiety were reported which persisted at six-month follow-up.

After ‘Mental/cognitive functions’ the most common target for interventions were the WHO function ‘Self-management’ [36,37,39,40,52,53,56,57,60] and ‘Activities of daily living [40,46,48,57,61]. A Canadian study with 25 low grade, high grade and meningioma patients who received Goal Management training found improved executive and daily functioning which persisted at four-month follow-up [57]. A later US study of an eight-week Resilient Living program was conducted with 62 low-grade glioma and meningioma patients. This psychosocial program developed resilience skills with an eight-week intervention - one individual or dyadic (patient/family caregiver) telehealth session of one hour duration; four online video modules of 5.5 hours over 8 weeks, and a daily journal. Participants found the intervention beneficial but time consuming [56]. Four studies targeted ‘Community & social life’ [37,39,47,53], ‘Carer & family support’ [37,49,50,56], two involved ‘Education and vocation’ [48,50], ‘Interpersonal interactions & relationships’ [37,39], one study with interventions for ‘Pain management’ and ‘Lifestyle modification’ [37]. Positive outcomes in cognitive performance and QoL persisted in three studies [41,53,57].

## Discussion

This scoping review highlights consistent evidence of significant, and frequently unmet rehabilitation need in adults diagnosed with a brain tumour, but also, promising findings of potential to benefit from targeted interventions. Most studies examined needs pertaining to mental/cognitive functions, and motor functions and mobility. Mental/cognitive functions can be significantly impacted after a brain tumour diagnosis and treatment, leading to high levels of psycho-oncologic need. People can experience potentially profound difficulties in memory and concentration, emotions, depression, anhedonia and poor quality of life. The impact extends to families and caregivers, whose needs for mental health supports are often unmet. Fatigue emerged as the most reported functional limitation alongside a range of motor function difficulties ranging from ataxia to motor weakness. While needs linked to mental/cognitive functions, and motor functions and mobility were frequently studied this may not reflect the reduced importance of other needs. This review found unmet rehabilitation needs were reported in a range of domains including physical, cognitive emotional, social, or information. Few studies investigated participation-level needs such as return to education/occupation or community and social life, and we recommend more focus on these areas in future research.

A key contribution of this review is the finding that needs do not depend on the type or grade of brain tumour and can be persistent over time. This raises significant implications for survivorship. The National Institute for Health and Care Excellence (NICE) guidelines for adults with brain tumours, recommended that rehabilitation should be considered at every stage of treatment and follow-up [63,64]. However, current clinical practice guidelines on rehabilitation approaches for brain tumours are inadequate [7]. Our research with healthcare professionals indicates that the absence of clear pathways and challenges in coordinating care can reduce access to rehabilitation services [65]. Clearer, clinical guidelines, for healthcare professionals/stakeholders, on best practice in rehabilitation are required. These should focus on increasing access to rehabilitation services and providing regular, ongoing care.

This review also describes the wide and varied range of interventions that have been investigated in primary studies, to improve function after a brain tumour diagnosis. The studies demonstrated feasibility of a wide range of intervention designs, from inpatient neuro-rehabilitation to outpatient and community-based exercise and cognitive interventions, some of which included families and carers. It was not the aim of this scoping review to determine effectiveness of any interventions, nor did we conduct quality assessment, so these findings are interpreted descriptively. Nonetheless, there was evidence of improved function and health-related quality of life after inpatient rehabilitation, and a range of benefits for community-based interventions, including crossover of benefit from physical activity interventions to cognitive and executive functions. From a feasibility perspective, studies indicated a need for supervision in exercise interventions. Unsupervised exercise tended to have poorer adherence than supervised sessions with cancer exercise specialists. This has implications for future intervention design and resourcing. It is also noteworthy that tailored cognitive interventions reported beneficial effects that often persisted over time.

Augmented reality (AR) studies are increasingly used to support patients in physical rehabilitation, but have had mixed findings, possibly due to the variation in design or perhaps the cognitive load of managing an immersive AR environment. A key contribution of this review were the benefits of multimodal rehabilitation, which utilised multiple therapies and a variety of approaches. These studies frequently addressed multiple WHO functions indicating the importance of multidisciplinary teams in addressing a range of unmet rehabilitation needs and enhancing patients’ quality of life. A focus on the optimal timing and duration of interventions by multidisciplinary teams in future research would be recommended.

The promising evidence from intervention studies has not fully translated into clinical practice [64,65]. In the post-acute setting, in a retrospective analysis of service utilisation by 719 patients diagnosed in Italy only 50% of patients received rehabilitation in the first six months post diagnosis and fewer than 10% of these patients received cognitive or language rehabilitation [54]. Variation in the provision of rehabilitation services has also been identified in the UK [64]. This may reflect a lack of awareness, among HCPs and patients/caregivers of the benefits of different types of rehabilitation for people after brain tumour diagnosis.

### Strengths and limitations

This scoping review used a systematic research methodology, including relevant search strategies and terms developed by the research team and included individuals with expertise in rehabilitation. A narrative review was undertaken to provide a useful synthesis of international evidence related to rehabilitation needs. However, the review had some limitations. No quality assessments were conducted and some studies had small sample sizes which may have resulted in difficulties with the reliability and generalisability of findings. Despite the social impact of a brain tumour diagnosis, a limited number of studies were conducted with caregivers/relatives. Also, the exclusion of conference abstracts may have caused some relevant studies to be omitted.

## Conclusion

The continuum of care for patients with brain tumours, from diagnosis to treatment to rehabilitation can involve many disciplines and supports. Reframing rehabilitation for people who have been diagnosed with brain tumour, with a focus on maintaining function, could help address unmet rehabilitation needs. This review contributes to evidence on needs and interventions for rehabilitation and can help inform the development of evidence based, high quality rehabilitation services. It identified areas for further research – more involvement of adults with a brain tumour and family/caregivers in their needs and priority for rehabilitation; more investment/resource in this area particularly in high demand areas and prioritising of intervention studies with promising findings towards implementation in practice.

## Supporting information

S1 Fig Frequency of reported tumours by type and sub-type. (TIF)

S1 Table Preferred Reporting Items for Systematic reviews and Meta-Analyses extension for Scoping Reviews (PRISMA-ScR) Checklist. (PDF)

S2 Table Search terms and strategy for databases. (PDF)

S3 Table Inclusion/exclusion criteria. (PDF)

S4 Table Characteristics of studies related to rehabilitation needs (n = 20). (PDF)

S5 Table Characteristics of intervention studies (n = 28). (PDF)

S6 Table Frequency of rehabilitation needs reported in studies (n = 20 papers). (PDF)

S7 Table Summary of types of interventions and WHO rehabilitation targets (n = 28). (PDF)
