# Supplementary figures and images for: Rehabilitation needs of adults after a brain tumour diagnosis: A scoping review
Source: PLoS One. 2025 Jul 17;20(7):e0325266. doi: 10.1371/journal.pone.0325266 (PMC12270154; doi:10.1371/journal.pone.0325266)

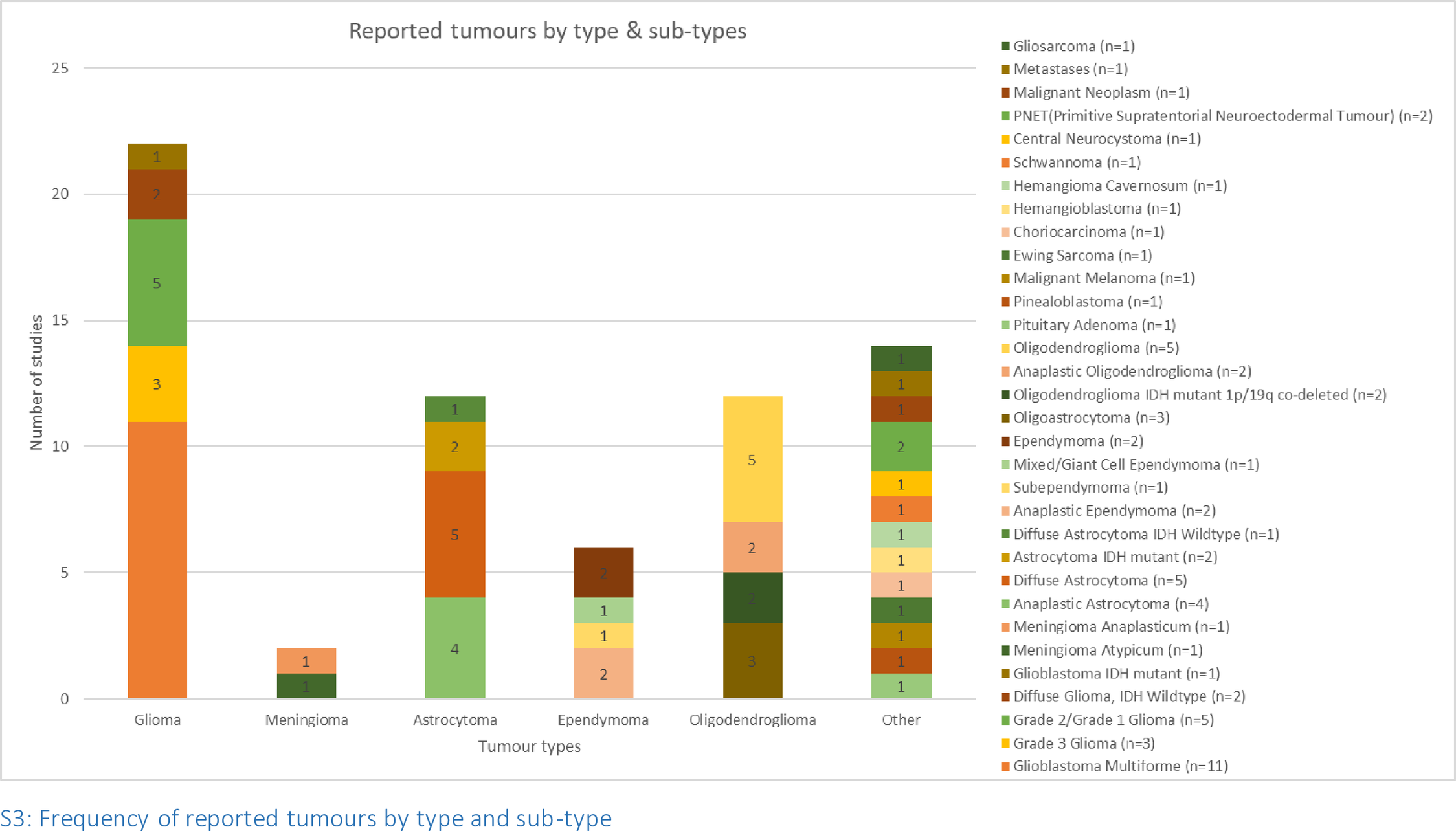

Supplement: S1 Fig — (TIF) [file pone.0325266.s001.tif]
