# Supplementary material for: Rehabilitation needs of adults after a brain tumour diagnosis: A scoping review
Source: PLoS One. 2025 Jul 17;20(7):e0325266. doi: 10.1371/journal.pone.0325266 (PMC12270154; doi:10.1371/journal.pone.0325266)
Supplement: S2 Table — (PDF) [file pone.0325266.s003.pdf]

S2 Table

S2: Search terms and strategy for databases

Databases<br>2003-2023	Search strategy
**EMBASE**<br>**Pubmed**<br>**CINAHL complete**<br>**PsychINFO**<br>**PEDro**	(MH "Brain Neoplasms+") OR (Brain tumo* OR brain cancer* OR brain Metastas* OR brain neoplasm* OR malignant brain tumo* OR cerebral cancer* OR cerebral tumo* OR Glioblastoma multiforme OR Glioma* OR ((Brain OR Cranial) AND (tumo* OR Neoplasm OR Cancer))
	AND
	“rehabilitation” OR “physical therapy” OR “physical outcomes” OR “behaviour therapy” OR “behavior therapy” OR “cognitive therapy” OR “cognitive outcomes” OR “functional outcomes” OR “rehabilitation intervention”
	OR
	"cognitive rehabilitation" OR "speech therapy" OR "neurocognitive rehabilitation" OR" neuropsychological rehabilitation" OR "non-pharmacological intervention" OR "health behavior change"
	AND
	“unmet needs” OR “rehabilitation needs” OR “patient* needs” OR “rehabilitation assessment” OR “rehabilitation care needs” OR “quality of life” OR “symptom burden”
