# Supplementary material for: Rehabilitation needs of adults after a brain tumour diagnosis: A scoping review
Source: PLoS One. 2025 Jul 17;20(7):e0325266. doi: 10.1371/journal.pone.0325266 (PMC12270154; doi:10.1371/journal.pone.0325266)
Supplement: S3 Table — (PDF) [file pone.0325266.s004.pdf]

S3: Inclusion/exclusion criteria

Criteria	Inclusion	Exclusion
**Population**	Aged 18 years or older<br>Confirmed diagnosis of brain tumour (WHO 2021 Classification of Central Nervous System tumours (a. Glioma, glioneuronal and neuronal tumour, ependymoma; b. Cranial nerve tumour; c. Meningioma))<br>Primary/secondary brain tumours	Aged 17 years or younger
**Intervention**	Neuro-rehabilitation services	Not applicable
**Outcomes**	Any patient outcomes related to rehabilitation care<br>Any patient outcomes related to rehabilitation interventions<br>Physical needs e.g. symptom burden, fatigue<br>Cognitive needs e.g. impaired memory/executive function<br>Psychosocial needs e.g. anxiety, depression, quality of life<br>Resultant rehabilitation needs (physical & cognitive)<br>Socioeconomic needs e.g. financial burden<br>Views of rehabilitation care – patients, HCPs, carers, families.	Studies testing the psychometric properties of patient health measures.
**Study Design**	Studies with empirical research methods (e.g. randomized control trials, case control studies, cohort studies, and cross-sectional studies)<br>Reviews	Non-empirical literature (e.g. opinion pieces, editorials)<br>Thesis/dissertations<br>Grey literature e.g. conference abstracts, reports etc.
**Reporting**	English language<br>Sufficient detail on unmet rehabilitation needs<br>Sufficient detail on results*	Not applicable
