# Supplementary material for: Rehabilitation needs of adults after a brain tumour diagnosis: A scoping review
Source: PLoS One. 2025 Jul 17;20(7):e0325266. doi: 10.1371/journal.pone.0325266 (PMC12270154; doi:10.1371/journal.pone.0325266)
Supplement: S4 Table — (PDF) [file pone.0325266.s005.pdf]

S~~upporting information file~~ 4 Table

S4: Characteristics of studies related to rehabilitation needs (n=20)

**Study (author & year, location) [ref]**	**Study population**	**Main findings**	**WHO functions/ categories 1-14***
Acquaye et al., 2017<br>USA<br>[15]	Patients with brain ependymoma<br>N=114	>Most common moderate-severe symptoms were fatigue (34%); difficulty with remembering (31%); disturbed sleep (29%); distress (27%); feeling drowsy (27%)<br>> Interference with work, activity, walking<br>>Reports of bodily pain; poor physical functioning; low vitality	1, 2, 6, 8, 10
Affronti et al., 2018<br>USA<br>[16]	Patients 6 months post low-grade glioma diagnosis<br>N=15	>Many experienced physical problems included fatigue (40%); memory/concentration (40%)<br>>Emotional problems included worry (60%); depression (40%); nervousness (47%); sadness (40%); loss of interest in usual activities (40%)<br>>Most frequent strategies were obtaining community support (87%); managing expectations (73%); support systems (67%); knowledge seeking about physical (67%) & behavioural (53%) symptoms	1, 13
Aprile et al., 2015<br>Italy<br>[17]	Outpatients with HGG<br>N=67	> 36.2% BFI ≥3 (clinically relevant fatigue)<br>Fatigue linked to QoL -physical functioning (p<0.01) & Emotional well-being (p<0.02)	1, 6
Benz et al., 2018<br>USA<br>[18]	Patients with intracranial meningioma<br>N=1722	>Overall significant decreases in QoL<br>> Lower levels of physical, emotional & mental health functioning vs controls<br>> Main differences in 4 domains: Physical & Social Functioning; Role-Physical; Role-Emotional & Vitality	1, 6, 11
Boele et al., 2015<br>The Netherlands<br>[19]	Patients with stable LGG<br>N=65	> Stable HRQOL for majority but 9%-12% declined in aspects of physical HRQOL<br>> Physical health declined long-term assessment vs mid-term assessment (P < .001)<br>>Physical functioning subscale significantly worse at longterm follow-up (P < .001)<br>>Physical HRQOL significantly worse at long-term versus (vs) midterm follow-up (both P < .001)<br>>Overall HRQOL scores improved/remained stable for 48%; decreased for 38.5%	1
Cantisano et al., 2021<br>France<br>[20]	Adult survivors of primary brain tumour<br>N=80	>More executive function (EF) problems in pt group than control<br>> High level of agreement between pt & caregivers' ratings<br>> Persistent EF effects post-treatment	1

		>Significant differences between pt vs control in emotional control (p=0.04); working memory (p=0.0007); task monitor (p=0.01); Metacognition index (MCI) (p=0.03) & Global Executive Composite (GEC) (p=0.02)	
Halkett et al., 2022<br>Australia<br>[21]	Patients with high grade glioma (HGG)<br>N=116	> Four distress trajectory groups - consistent low distress (18%), low-high distress (38%), high-low distress (24%) & consistent high distress (19%)<br>>Younger pts report decreased distress; older pts report high distress<br>>Less education, lower physical wellbeing, more unmet needs, but higher functional wellbeing in high distress group vs compared to low-high group	1
Kearney et al., 2022<br>Australia<br>[22]	Patients with left-hemisphere tumours<br>N=37	> Higher than average problems associated with aphasia - perceived disability (mean 11.76); impact (mean 13.22)<br>> Depression (27%), anxiety (43%), stress(14%) above subclinical levels	1
Khan et al., 2013<br>Australia<br>[23]	Patients with WHO central nervous system tumours<br>N=106	>Good functional recovery in majority<br>> Over half reported pain (56%), of which headaches (42%); ataxia (44%); seizures (43%); paresis (37%); cognitive dysfunction (36%) & visual impairment (35%)<br>>High levels of depression (≈20%)<br>>Age, recent diagnoses, aggressive tumour types & pain associated with poor function & well-being	1, 2, 6
Kim et al., 2012<br>Korea<br>[24]	Patients with resected brain tumors<br>N=25	>Fatigue linked to KPS, MBI, & EORTC QLQ-C30 physical functioning & insomnia scales<br>> Insomnia a predictor of fatigue before rehab; fatigue & physical functioning scales NB predictors of fatigue after rehab	1, 6
Krajewski et al., 2023<br>Poland<br>[25]	Patients malignant (N=26) & non-malignant (N=66) primary brain tumors<br>N=92	>Similar postoperative complications & loss of independent gait across groups (~30%)<br>>More paralysis & paresis in malignant group (p < 0.001)<br>>Both groups have similar rehab needs<br>> Worse functional outcomes in malignant tumor group did not affect LoS or rehabilitation	6, 8
Kvale et al., 2009<br>USA<br>[26]	GBM patients<br>N=50	> Significant correlations between distress score & social/family well-being (P=0.001) and emotional well-being EWB (P=0.001)<br>>Assessment of distress clinically relevant	1
Lowe et al., 2014<br>Canada<br>[27]	Patients with brain metastases<br>N=31	>Sedentary behaviour associated with better physical functioning but decreased psychosocial functioning	1, 6

Miklja et al; 2022<br>USA<br>[28]	Patients with low/high-grade glioma<br>N=38	> Low pre-morbid physical activity levels =more distress in HRQOL, sleep & fatigue domains	1, 7
Pace et al., 2016<br>Italy<br>[29]	Patients with primary brain tumour<br>N=719	>Limited rehab interventions during first year after diagnosis, confirming rehab needs largely unmet<br>>Inpatient rehab (n=92); outpatient personalized program with intensive rehab plan (n=22); outpatients rehab interventions (n=85)	1, 6
Piil et al., 2017<br>Denmark<br>[30]	Patients with HGG & caregivers<br>N=63<br>(30 pts & 33 caregivers)	> Physical activity decreased over time, 89% of patients active < 3 hr/week<br>> Improved anxiety (p = .0095)<br>> Significant improvement in emotional well-being (p = .0023)<br>>Pt needs included promoting physical activities, psychological symptom management strategies & life planning<br>> Carers' needs included supportive care, education, information & rehab	1, 6
Porensky et al., 2013<br>[31]	Patients with brain tumour<br>N=116<br>(58 high-grade, 58 low-grade)	> No differences between high & low grade for distress/QoL<br>>Common sources of distress=forgetfulness/memory problems (49%), fatigue/ lack of energy (47%), difficulty concentrating (34%), worry (30%) & feeling drowsy (30%)<br>>Emotional (p =.001) & cognitive concerns (p =.001) significantly predicted decreased QOL	1, 6
Reinert et al., 2020<br>Germany<br>[32]	Patients with brain tumour & care-givers<br>N=44	>31.9% of pts clinically depressed<br>>Psycho-oncologic need positively associated with mild (p = 0.001) or moderate-severe (p < 0.001) depression<br>> Pt information need associated with depression (p = 0.022)	1, 13, 14
Rimmer et al., 2023<br>UK<br>[33]	Patients with lower-grade gliomas (LGG), N=28	> Diverse & substantial self-management strategies<br>>Most used strategy types 'using support' (n=28), 'creating a healthy environment' (n=28), 'meaning making' (n=27) & 'self-monitoring' (n=27)<br>>Most used strategies 'accepting the tumour and its consequences' (n=26), 'receiving support from friends (n=24) and family' (n=24) & 'reinterpreting negative consequences' (n=24)	13
Umezaki et al., 2020<br>Japan<br>[34]	Patients with WHO grade II-IV glioma<br>N=76	>Seven symptoms occurred in more than 50% - fatigue, future uncertainty, drowsiness, communication deficit, financial difficulties, motor dysfunction & weakness of legs<br>>Symptoms & neurological issues associated with poor QoL	1, 6, 8

*WHO package of interventions for rehabilitation: module 7: malignant neoplasms; vs=versus; UE=upper-extremity; ADL=activities of daily living; Tx=treatment; IPR=inpatient rehabilitation program; NR=not reported; PT= physical therapist; Min=minutes; [ref]=reference
