# Supplementary material for: Rehabilitation needs of adults after a brain tumour diagnosis: A scoping review
Source: PLoS One. 2025 Jul 17;20(7):e0325266. doi: 10.1371/journal.pone.0325266 (PMC12270154; doi:10.1371/journal.pone.0325266)
Supplement: S5 Table — (PDF) [file pone.0325266.s006.pdf]

S5 Table

S5: Characteristics of intervention studies (n=28)

**Study (author & year) [ref]**	**Study population**	**Rehabilitation intervention**	**Main findings**	**WHO functions/ categories 1-14***
Baima et al., 2017<br>NR<br>[35]	Patients with high-grade brain tumors<br>N=15	Novel strength & balance home exercise program (duration one month);<br>one time training includes exercise video & live demonstration of resistance band exercises, a balance exercise, &recommendations for walking	> 60% continued with exercises for 1 month<br>>Higher exercise frequency associated with married, high income patients with above average scores on physical (p=0.047) & emotional well-being (p=0.054)<br>> Patients with higher QoL at baseline more likely to persist with exercise after study (p=0.018)	7
Boele et al., 2018<br>The Netherlands<br>[36]	Adult glioma patients<br>N=89	Online guided self-help intervention for depressive<br>Symptoms.3 groups: glioma intervention (GI) group, waiting list group (GWL)& control group with intervention directly following assessments/after 12 weeks interval adaptation of 5 week online guided self-help course founded on PST principles	>Decreased fatigue in GI vs GWL group<br>>No significant differences between groups in depression	1, 13
Clarke et al., 2013<br>USA<br>[37]	Patients with advanced cancer & caregivers<br>N=131	Multidisciplinary intervention (RCT)<br>66-session, 90 mins, structured, intervention arm or standard care, followed by 10 brief telephone counseling sessions over next 6 months (mths)<br>addressed 5 domains of quality of life (QOL): cognitive, physical, emotional, social, & spiritual	>QOL higher in intervention v control (P = .02)<br>>no effect on QOL for 10 brief telephone contacts<br>>No intervention effect on caregiver QOL	1, 2, 9, 11, 12, 13, 14

		education, cognitive behavioral coping strategies, open discussion, support & 15-minute deep breathing/guided imagery relaxation segment<br>physical therapist, psychologist/psychiatrist, advanced practice nurse, certified hospital chaplain/clinical social worker		
Culos-Reed et al., 2017<br>Canada<br>[38]	HGG patients<br>N=16	Feasibility of an exercise study<br>- exercise assessments (incremental cycling exercise test & hand grip), PA & QoL at 3 time points<br>- T1 prior to starting Temozolamide chemotherapy with radiation, T2 at 2 months & T3 at 8 months	>Preference for moderate, unsupervised exercise at home<br>>Less than a quarter (<25%) met PA guidelines<br>>At 2 months, PA minutes associated with well–being ($p<0.05$)	6, 7
Dahlberg et al., 2022<br>NR<br>[39]	Survivors with brain tumors & caregivers<br>N=19	Testing of social network-mapping tool CareMaps	> Positive opinions about CareMaps but questions about design & use - how to use/when best time to use	9, 11, 13
Fahrenholtz et al., 2019<br>Denmark<br>[40]	Primary glioma (male)<br>N=5	Physical therapy & occupational therapy–based intervention vs usual rehab care<br>6 weeks group physical therapy with focus on exercise (cardiovascular training, resistance training)<br>If ADL difficulties client- centred occupational therapy intervention	>Use problem-solving coping strategies<br>>Also passive & emotion-focused strategies related to confronting diagnosis<br>>Intervention's impact on HRQoL can increase pts' resources	1, 6, 8, 13
Fouda et al., 2023<br>USA<br>[41]	Patients post surgical resection of	Evaluation of Cogmed Working Memory Training (CWMT) program	> Significant improvement in all post-CWMT neurocognitive function<br>>Improvement maintained at 3 month follow-up	1

	WHO grade I intracranial meningioma<br>N=4	- intensive, hierarchical & systematic training<br>- 25 online training sessions for 5 weeks (each 30–45 mins)<br>-		
Gehring et al., 2009<br>Netherlands<br>[42]	Patients with low-grade anaplastic gliomas<br>N=140	Evaluation of cognitive rehabilitation program (CRP)<br>both computer-based attention retraining & compensatory skills training of attention, memory & executive functioning	> Positive effects on short-term cognitive complaints & longer-term cognitive performance & mental fatigue<br>> Improved subjective cognitive functioning & perceived burden (post intervention)	1
Gehring et al., 2020<br>Netherlands<br>[43]	WHO grades II/III glioma<br>N=34	Exercise intervention (RCT), exercise group vs active control<br>homebased, coached remotely, over 6 months<br>3 aerobic exercise sessions per week (20–45 mins), moderate to vigorous intensity	>Better outcomes in exercise group on self-reported cognitive symptoms, fatigue, sleep, mood & HRQoL	1, 7
Gildea et al., 2020<br>Australia<br>[44]	Adults with primary brain cancer<br>N=12	Individualised exercise intervention<br>18-week, weekly exercise goal ≥150-mins of moderate-intensity exercise including ≥2 resistance training sessions	>No exercise-related serious adverse events (SAE); recruitment (80%), retention (92%) & adherence (83%)<br>>Significant improvements in QOL (p= 0.03), fatigue (p= 0.04), physical activity (P=0.01), aerobic fitness (P=0.01), physical function (P=0.04) & lower body strength (P<0.001)	1, 6
Hansen et al., 2018<br>Denmark<br>[45]	Patients with glioma<br>N=24	Interdisciplinary rehabilitation intervention<br>Part 1 outpatient therapeutic supervised training (6 weeks) & Part 2 unsupervised training in a local gym (6 weeks)	>High feasibility in part 1 safety (100%), consent rate (>80%), drop-out (<20%), adherence (>80%) & patient satisfaction (>80%)<br>> Failed feasibility in part 2 low adherence (54%), high drop-out (45%) & low satisfaction (62.5%)<br>>Significant increased strength in leg press (32%), knee extension (17%), knee flexion (15%), arm flexion (25%), arm extension (26%) & reduced step frequency in 10 meter walk	6, 7
Hansen et al., 2020	Patients with gliomas	Supervised rehabilitation	>Better QoL in intervention group vs control	1, 6, 7, 8

Denmark<br>[46]	N=64	physical therapy & occupational therapy-based interventions compared to usual rehab	>Better self-rated secondary outcomes - cognitive functioning, fatigue & aerobic power (objectively measured)	
Maialetti et al., 2020<br>Italy<br>[47]	Brain tumor-related epilepsy pts<br>N=33	Multimodal rehabilitation pathway (MRP)<br>epileptological follow-up, supportive meeting groups, social assistance; patients with cognitive deficits could also obtain 12-week neurocognitive training	>MRP improved seizure control, cognitive performances, social relations, patients perceived support & QoL (related to cognitive efficacy)<br>>Improved cognition (=.04) & significant decrease in cognitive related distress (P =.04)	1, 11
McCarty et al., 2017<br>USA<br>[48]	Adult pts with malignant brain tumors<br>N=49	Interdisciplinary outpatient rehabilitation program<br>at least 2/3therapies (PT, OT, & SLP), from 2 half days per wk (3 hrs/d) to 5 full days per wk (6 hrs/d), other services<br>included nursing & physician care, vocational rehab & psychology counselling, at six affiliated day rehab sites for 35 months	> Negative associations between overall well-being & depression (all P< .0001)<br>> Positive correlation between Day Rehabilitation Outcome Scale (DayROS) & overall well-being FACT-Br (P=.0058); negative association between DayROS & pain (P=.028)	1, 6, 8, 10
Milbury et al., 2018<br>USA<br>[49]	HGG pts<br>N=10<br>(5 pts, 5 caregivers)	Dyadic yoga program (DYP) for pts & caregivers<br>12 sessions that focus on breathing exercises, gentle movements & guided meditations	>Reductions in sleep disturbances<br>>Significant improvements in pt & caregiver QOL	1, 7, 14
Nordentoft et al., 2022<br>Denmark<br>[50]	Patients with high-grade glioma<br>N= 33 (17 pts, 16 carers)	Evaluation of REHPA-HGG<br>4-day tailored residential programme with 2-day follow-up programme 12 weeks later	>REHPA-HGG high in feasibility relevance & satisfaction<br>> Qualitative findings highlight value of peer interactions, tailored information for individuals & acceptance of unpredictability in future (linked to difficulties integrating action plans)	1, 6, 10, 14

Ooi et al., 2013<br>Malaysia<br>[51]	Patients with primary intracranial tumours<br>N=38	All patients received some form of rehabilitation interventions after surgery	> Concurrent improvements in function & health related quality of life (HRQoL)<br>>Greatest increase in emotional functioning (p=0.003)<br>>Reduced symptom burden (fatigue, nausea, vomiting, pain, headache); also less future uncertainty (p<0.05)<br>>Relationship between functional status & HRQoL global health status	1, 6
Ownsworth et al., 2015<br>Australia<br>[52]	Patients with primary brain tumour<br>N=50	Home-based psychosocial intervention Making Sense of Brain Tumor (MSoBT) program<br>Immediate treatment (trt) group received 10-session MSoBT program, waitlist group received usual care for 10 weeks, re-assessed then received MSoBT program	>Lower levels of depression in immediate treatment group<br>>Higher levels of functional well-being & global QOL in immediate treatment group<br>> Effects persisted at 6 mth follow-up	1
Ownsworth et al., 2023<br>Australia<br>[53]	Adults with primary brain tumour<br>(N=118)<br>82 pts & 36 caregivers	Remote delivery of MAST (Tele-MAST) via telephone & videoconferencing (Making Sense of Brain Tumor)<br>Tele-MAST intervention v standard care<br>10 1-h sessions per week (3 core & 7 tailored) via Zoom videoconferencing<br>Interventions included psychoeducation on emotional /cognitive changes; psychotherapy to address low mood/anxiety & strategy training to manage subjective cognitive effects	> Lower depression Tele-MAST group (p=0.002) & 6-wks later (p=0.010) vs standard care<br>> Significantly better global QoL, emotional QoL & lower anxiety Tele-MAST group	1, 11, 13
Pace et al., 2007<br>Italy<br>[54]	Patients with malignant brain tumor<br>N=121	Post-discharge rehab home care included neuro rehabilitation	>Functional gain Barthel Index improved in 47 (39%) pts<br>> Majority (72%) improved in at least 1 QoL domain	1, 6

Pieczyńska et al., 2023<br>Poland<br>[55]	Adult pts with HGG<br>N=47	Augmented reality based rehabilitation exercises<br>control group received standard recommendations regarding minimum level of physical activity<br>Experimental group had supervised exercises during 30-day RT treatment then exercises at home using Neuroforma remote program	> Significant decrease in hand grip strength (HGS) and attention for control group	1, 6, 7
Rhudy et al., 2023<br>USA<br>[56]	Patients with acquired brain disorders/stroke & caregivers<br>N=16<br>8 pts & 8 caregivers	Resilient Living program, psychosocial intervention with focus on building resilience skills	> Participants found intervention useful & flexibile but finding time to engage was challenge<br>> Further research needed for optimal timing of resilience interventions	1, 6, 13, 14
Richard et al., 2019<br>Canada<br>[57]	Primary brain tumor survivors<br>N=25	Evaluated of Goal Management Training (GMT) (RCT)<br>3 groups; GMT group, active control Brain Health Program (BHP), or a wait-list (WAIT) usual care control group<br>GMT=sessions with added brain tumour-specific content (e.g., tumour or treatment effects on cognitive functioning)<br>BHP=content about brain & cognition but no cognitive strategy training, and served as an active comparison condition.<br>Both interventions delivered in eight weekly 2-hr sessions by clinical neuropsychologist with homework between sessions<br>GMT homework between sessions=	>Executive functions improved with GMT group (post-training P=0.077, follow-up P=0.046)<br>>Both intervention groups fewer cognitive concerns at post-training (P=0.049) & follow-up (P<0.001)<br>>Greatest functional goal attainment in GMT group (post-training P=0.027, follow-up P=0.064)<br>>Persistent effects for GMT group	1, 8

		monitoring cognitive problems & applying learned strategies to day-to-day activities<br>BHP homework =“brain challenges” (e.g., crossword puzzles), attention to factors supporting general brain health (e.g., sleep hygiene, balanced diet, physical exercise) & stress reduction strategies		
Spencer et al., 2021<br>USA<br>[58]	Pts with high-grade glioma<br>N=30	Evaluation of 10-week exercise intervention<br>3 groups: exercise group (weekly in-person exercise classes), usual care group (normal daily activities) & education group (60-min education session & self-reported exercise log)	>Less fatigue (–46% change visual analog fatigue scale (VAFS) & improved QOL in exercise group vs other groups<br>> High attendance (80%) for exercise group	1, 7
Troschel et al., 2020<br>Germany<br>[59]	Pts with brain tumours & relatives<br>N=15	Evaluation of ski exercise intervention<br>week-long daily ski sessions with professional ski guides (also dedicated physicians)	> Increase in activity & QOL<br>>Decrease in distress during intervention	1, 7
Van der Linden et al., 2021<br>NR<br>[60]	Low-grade glioma & meningioma<br>N=62	Evaluation of tablet-based cognitive rehabilitation program (ReMind)<br>10 week eHealth app ReMind, face-to-face, psychoeducation, strategy-training & attention retraining	>90% rated program as ‘good’ or ‘excellent’<br>>All found tablet-app suitable for cognitive rehabilitation	1, 13
Yoon et al., 2015<br>Korea<br>[61]	Brain tumour pts with upper-extremity (UE) dysfunction<br>N=40	Evaluation of virtual reality-based rehabilitation (RCT)<br>VR program for intervention group 9 sessions (30 mins per session) & 6 sessions of conventional OT (30 mins per session) for 3 weeks;	> Combined virtual reality-based rehab & OT may be more effective than OT<br>>Improvements in shoulder/elbow/forearm function in intervention group & hand function in control group<br>>Intervention group - Box and Block Test (p=0.044); Manual Function Test (p=0.007) & Fugl-Meyer (p=0.012) vs control	6, 8

		conventional OT alone for control group 15 sessions (30 mins per session) for 3 weeks	>Improved activities of daily living (ADL) in both groups; intervention (p=0.005), control (p=0.0011)	
Zucchella et al., 2013<br>Italy<br>[62]	Pts with primary brain tumours<br>N=58	Evaluation of early cognitive rehabilitation<br>Rehab group 16 one-hour individual sessions, therapist-guided cognitive training, over 4 weeks, combining computer exercises & metacognitive training<br>control group received usual care without cognitive training	> Significant improvement of cognitive functions – mostly visual attention & verbal memory	1

*WHO package of interventions for rehabilitation: module 7: malignant neoplasms; Pts=patients; FACT-Br=Functional Assessment of Cancer Therapy–Brain; DayROS=Day Rehabilitation Outcome Scale; HRQoL=health-related quality of life; mt=month; wk=weeks;[ref]=reference
