# Supplementary material for: Rehabilitation needs of adults after a brain tumour diagnosis: A scoping review
Source: PLoS One. 2025 Jul 17;20(7):e0325266. doi: 10.1371/journal.pone.0325266 (PMC12270154; doi:10.1371/journal.pone.0325266)
Supplement: S6 Table — (PDF) [file pone.0325266.s007.pdf]

S6 Table

S6: Frequency of rehabilitation needs reported in studies (n = 20 papers)

Paper	Physical needs	Cognitive needs	Psychosocial/emotional	QOL – needs related to daily living	Information needs
Acquaye et al., 2017 [15]	√	-	√	√	-
Affronti et al., 2018 [16]	√	√	√	√	-
Aprile et al., 2015 [17]	√	-	√	√	-
Benz et al., 2018 [18]	-	-	-	√	-
Boele et al., 2015 [19]	-	-	-	√	-
Cantisano et al., 2021 [20]	-	√	-	-	-
Halkett et al., 2022 [21]	√	√	√	-	-
Kearney et al., 2022 [22]	-	√	√	-	-
Khan et al., 2013 [23]	√	√	-	√	-
Kim et al., 2012 [24]	√	-	√	√	-
Krajewski et al., 2023 [25]	√	-	-	√	-
Kvale et al., 2009 [26]	-	-	√	√	-
Lowe et al., 2014	√	-	-	√	-

[27]					
Miklja et al; 2022 [28]	**√**	**-**	**-**	**√**	**-**
Pace et al., 2016 [29]	**√**	**√**	**-**	**-**	**-**
Piil et al., 2017 [30]	**√**	**-**	**√**	**√**	**-**
Porensky et al., 2013 [31]	**√**	**√**	**-**	**√**	**-**
Reinert et al., 2020 [32]	**-**	**-**	**√**	**-**	**√**
Rimmer et al., 2023 [33]	**-**	**√**	**√**	**-**	**-**
Umezaki et al., 2020 [34]	**√**	**-**	**-**	**-**	**√**

√ denotes corresponding need is reported; - denotes corresponding need is not reported; QOL=quality of life
