# Supplementary material for: Rehabilitation needs of adults after a brain tumour diagnosis: A scoping review
Source: PLoS One. 2025 Jul 17;20(7):e0325266. doi: 10.1371/journal.pone.0325266 (PMC12270154; doi:10.1371/journal.pone.0325266)
Supplement: S7 Table — (PDF) [file pone.0325266.s008.pdf]

S~~upporting information file~~ 7 Table

S7: Summary of types of interventions and WHO rehabilitation targets (n=28)

| ***Authors [ref]*** | ***Rehab intervention*** | ***Rehab target WHO categories 1-14**** | | | | | | | | | | | | | |
|---|---|---|---|---|---|---|---|---|---|---|---|---|---|---|---|
| | | **1.** | **2.** | **3.** | **4.** | **5.** | **6.** | **7.** | **8.** | **9.** | **10.** | **11.** | **12.** | **13.** | **14.** |
| Baima et al., 2017 [35] | Strength & balance exercise program (home based) | - | - | - | - | - | - | √ | - | - | - | - | - | - | - |
| Boele et al., 2018 [36] | Online self-help intervention for depressive symptoms | √ | - | - | - | - | - | - | - | -- | - | - | - | - | - |
| Clarke et al., 2013 [37] | Multidisciplinary intervention | √ | √ | - | - | - | | | | √ | - | √ | √ | √ | √ |
| Culos-Reed et al., 2017 [38] | Exercise study | - | - | - | - | - | √ | √ | - | - | - | - | - | - | - |
| Dahlberg et al., 2022 [39] | Social network-mapping tool CareMaps | - | - | - | - | - | - | - | - | √ | - | √ | - | √ | - |
| Fahrenholtz et al., 2019 [40] | Physical therapy & occupational therapy | √ | - | - | - | - | √ | - | √ | - | - | - | - | √ | - |
| Fouda et al., 2023 [41] | Cogmed Working Memory Training (CWMT) | √ | - | - | - | - | - | - | - | - | - | - | - | - | - |
| Gehring et al., 2009 | Evaluation of cognitive | √ | - | - | - | - | √ | - | - | - | - | - | - | - | - |

| | | | | | | | | | | | | | | | |
|---|---|---|---|---|---|---|---|---|---|---|---|---|---|---|---|
| [42] | rehabilitation program (CRP) | | | | | | | | | | | | | | |
| Gehring et al., 2020 [43] | Exercise intervention | √ | - | - | - | - | √ | √ | - | - | - | - | - | - | - |
| Gildea et al., 2020 [44] | Individualised exercise intervention | √ | - | - | - | - | √ | - | - | - | - | - | - | - | - |
| Hansen et al., 2018 [45] | Interdisciplinary rehabilitation | - | - | - | - | - | √ | √ | - | - | - | - | - | - | - |
| Hansen et al., 2020 [46] | Supervised physical & occupational therapy vs usual rehab | √ | - | - | - | - | √ | √ | √ | - | - | - | - | - | - |
| Maialetti et al., 2020 [47] | Multimodal rehabilitation pathway (MRP) | √ | - | - | - | - | - | - | - | - | - | √ | - | - | - |
| McCarty et al., 2017 [48] | Interdisciplinary outpatient rehabilitation | √ | - | - | - | - | √ | - | √ | - | √ | - | - | - | - |
| Milbury et al., 2018 [49] | Dyadic yoga program (DYP) | √ | - | - | - | - | - | √ | - | - | - | - | - | - | √ |
| Nordentoft et al., 2022 [50] | Multimodal rehabilitation palliative care (REHPA-HGG) | √ | - | - | - | - | - | - | - | - | - | - | - | - | - |
| Ooi et al., 2013 [51] | Some rehabilitation after surgery | √ | - | - | - | - | - | - | - | - | - | √ | - | √ | - |
| Ownsworth et al., 2015 [52] | Making Sense of Brain | √ | - | - | - | - | - | - | - | - | - | - | - | - | - |

| | | | | | | | | | | | | | | | |
|---|---|---|---|---|---|---|---|---|---|---|---|---|---|---|---|
| | Tumor (MSoBT) home-based | | | | | | | | | | | | | | |
| Ownsworth et al., 2023 [53] | MAST (Tele-MAST) (Making Sense of Brain Tumor) – remote delivery | √ | - | - | - | - | - | - | - | - | - | √ | - | √ | - |
| Pace et al., 2007 [54] | Post-discharge rehabilitation (home care) | √ | - | - | - | - | - | - | - | - | - | - | - | - | - |
| Pieczyńska et al., 2023 [55] | Augmented reality based rehabilitation exercises | √ | - | - | - | - | √ | √ | - | - | - | - | - | - | - |
| Rhudy et al., 2023 [56] | Resilient Living program | √ | - | - | - | - | √ | - | - | - | - | - | - | √ | √ |
| Richard et al., 2019 [57] | Goal Management Training (GMT) | √ | - | - | - | - | - | √ | - | - | - | - | - | - | - |
| Spencer et al., 2021 [58] | 10-week exercise intervention | √ | - | - | - | - | - | - | - | - | - | - | - | √ | - |
| Troschel et al., 2020 [59] | Ski exercise intervention | √ | - | - | - | - | √ | - | √ | - | - | - | - | - | - |
| Van der Linden et al., 2021 [60] | Cognitive rehabilitation program (ReMind) | √ | - | - | - | - | - | - | - | - | - | - | - | - | - |
| Yoon et al., 2015 [61] | Virtual reality-based rehabilitation | - | - | - | - | - | √ | | √ | - | - | - | - | - | - |

| | | | | | | | | | | | | | | | |
|---|---|---|---|---|---|---|---|---|---|---|---|---|---|---|---|
| Zucchella et al., 2013 [62] | Cognitive rehabilitation | √ | - | - | - | - | - | - | - | - | - | - | - | - | - |

*WHO functions 1-14: 1=Mental/cognitive functions; 2=Pain management; 3=Bowel and bladder management; 4=Sexual functions & intimate relationships; 5=Cardiovascular and immunological functions, 6=Motor functions and mobility; 7=Exercise and fitness; 8=Activities of daily living; 9 Interpersonal interactions & relationships; 10=Education and vocation, 11= Community & social life; 12=Lifestyle Modification; 13=Self-management; 14=Carer & family support; Rehab=rehabilitation; √ denotes corresponding rehabilitation target is addressed; - denotes corresponding target is not addressed

S3: Characteristics of eligible studies continued (n=48) - how is this different from above and table S2. Tables and legends should be self explanatory and standalone – why is there only 5 studies and then 48??

| ***Authors (6-10)*** | ***No of participants*** | ***Type of tumour*** | ***Rehabilitation intervention*** | ***Rehabilitation target 1-5 (WHO interventions for rehabilitation in cancer)*** | | | | |
|---|---|---|---|---|---|---|---|---|
| | | | | 1.Mental/cognitive functions | 2.Pain management | 3.Bowl and bladder management | 4.Sexual functions & intimate relationships | 5.Cardiovascular and immunological functions |
| Fahrenholtz et al., 2019 | N=5 | Men, with primary glioma | Physical therapy & occupational therapy | **X** | **0** | **0** | **0** | **0** |
| Fouda et al., 2023 | N=4 | Patients post surgical resection of WHO grade I intracranial meningioma | Cogmed Working Memory Training (CWMT) | **X** | **0** | **0** | **0** | **0** |
| Gehring et al., 2009 | N=140 | Patients with low-grade anaplastic gliomas | Evaluation of cognitive rehabilitation program (CRP) | **X** | **0** | **0** | **0** | **0** |
| Gehring et al., 2020 | N=34 | Clinically stable patients with WHO grades II/III glioma | Exercise intervention | **X** | **0** | **0** | **0** | **0** |
| Gildea et al., 2020 | N=12 | Adults with primary brain cancer, post-radiotherapy | Individualised exercise intervention | **X** | **0** | **0** | **0** | **0** |

| **Rehabilitation target continued 6-14 (WHO interventions for rehabilitation in cancer)** | | | | | | | | |
|---|---|---|---|---|---|---|---|---|
| 6.Motor functions and mobility | 7.Exercise and fitness | 8.Activities of daily living | 9.Interpersonal interactions & relationships | 10.Education and vocation | 11.Community & social life | 12.Lifestyle modification | 13.Self-management | 14.Carer & family support |
| **X** | **0** | **X** | **0** | **0** | **0** | **0** | **X** | **0** |
| **0** | **0** | **0** | **0** | **0** | **0** | **0** | **0** | **0** |
| **X** | **0** | **0** | **0** | **0** | **0** | **0** | **0** | **0** |
| **X** | **X** | **0** | **0** | **0** | **0** | **0** | **0** | **0** |
| **X** | **0** | **0** | **0** | **0** | **0** | **0** | **0** | **0** |

| *Authors (11-17)* | *No of participants* | *Type of tumour* | *Rehabilitation intervention* | *Rehabilitation target 1-5 (WHO interventions for rehabilitation in cancer)* | | | | |
|---|---|---|---|---|---|---|---|---|
| | | | | 1.Mental/cognitive functions | 2.Pain management | 3.Bowl and bladder management | 4.Sexual functions & intimate relationships | 5.Cardiovascular and immunological functions |
| Hansen et al., 2018 | N=24 | Patients with glioma | Interdisciplinary rehabilitation | **0** | **0** | **0** | **0** | **0** |
| Hansen et al., 2020 | N=64 | Patients with gliomas | Supervised physical & occupational therapy vs usual rehab | **X** | **0** | **0** | **0** | **0** |
| Maialetti et al., 2020 | N=33 | Brain tumor-related epilepsy patients | Multimodal rehabilitation pathway (MRP) | **X** | **0** | **0** | **0** | **0** |
| McCarty et al., 2017 | N=49 | Adult patients with malignant brain tumors | Interdisciplinary outpatient rehabilitation | **X** | **0** | **0** | **0** | **0** |
| Milbury et al., 2018 | N=10 (5 pts, 5 caregivers) | HGG patients, newly diagnosed | Dyadic yoga program (DYP) | **X** | **0** | **0** | **0** | **0** |
| Nordentoft et al., 2022 | N= 33 (17 pts, 16 carers) | Patients with high-grade glioma | Multimodal rehabilitation palliative care (REHPA-HGG) | **x** | **0** | **0** | **0** | **0** |
| Ooi et al., 2013 | N=38 | Patients with primary intracranial tumours | Some rehabilitation after surgery | **x** | **0** | **0** | **0** | **0** |

| **Rehabilitation target continued 6-14 (WHO interventions for rehabilitation in cancer)** | | | | | | | | |
|---|---|---|---|---|---|---|---|---|
| 6.Motor functions and mobility | 7.Exercise and fitness | 8.Activities of daily living | 9.Interpersonal interactions & relationships | 10.Education and vocation | 11.Community & social life | 12.Lifestyle modification | 13.Self-management | 14.Carer & family support |
| **X** | **X** | **0** | **0** | **0** | **0** | **0** | **0** | **0** |
| **X** | **X** | **X** | **0** | **0** | **0** | **0** | **0** | **0** |
| **0** | **0** | **0** | **0** | **0** | **X** | **0** | **0** | **0** |
| **X** | **0** | **X** | **0** | **X** | **0** | **0** | **0** | **0** |
| **0** | **X** | **0** | **0** | **0** | **0** | **0** | **0** | **X** |
| **X** | **0** | **0** | **0** | **X** | **0** | **0** | **0** | **X** |
| **X** | **0** | **0** | **0** | **0** | **0** | **0** | **0** | **0** |
| **0** | **0** | **0** | **0** | **0** | **0** | **0** | **0** | **0** |
| **0** | **0** | **0** | **0** | **0** | **X** | **0** | **X** | **0** |

| ***Authors (18-25)*** | ***No of participants*** | ***Type of tumour*** | ***Rehabilitation intervention*** | ***Rehabilitation target 1-5 (WHO interventions for rehabilitation in cancer)*** | | | | |
|---|---|---|---|---|---|---|---|---|
| | | | | 1.Mental/cognitive functions | 2.Pain management | 3.Bowl and bladder management | 4.Sexual functions & intimate relationships | 5.Cardiovascular and immunological functions |
| Ownsworth et al., 2015 | N=50 | Patients with primary brain tumour | Making Sense of Brain Tumor (MSoBT) home-based | **X** | **0** | **0** | **0** | **0** |
| Ownsworth et al., 2023 | N=118 82 pts & 36 caregivers | Adults with primary brain tumour | MAST (Tele-MAST) (Making Sense of Brain Tumor) – remote delivery | **X** | **0** | **0** | **0** | **0** |
| Pace et al., 2007 | N=121 | Patients with malignant brain tumor | Post-discharge rehabilitation (home care) | **X** | **0** | **0** | **0** | **0** |
| Pieczyńska et al., 2023 | N=47 | Adult patients with HGG | Augmented reality based rehabilitation exercises | **X** | **0** | **0** | **0** | **0** |
| Rhudy et al., 2023 | N=16 8 pts & 8 caregivers | Patients with acquired brain disorders/stroke | Resilient Living program | **X** | **0** | **0** | **0** | **0** |
| Richard et al., 2019 | N=25 | Primary brain tumor survivors | Goal Management Training (GMT) | **X** | **0** | **0** | **0** | **0** |
| Spencer et al., 2021 | N=30 | Patients with high-grade glioma | 10-week exercise intervention | **X** | **0** | **0** | **0** | **0** |
| Troschel et al., 2020 | N=15 | Patients with brain tumours & relatives | Ski exercise intervention | **X** | **0** | **0** | **0** | **0** |

| **Rehabilitation target continued 6-14 (WHO interventions for rehabilitation in cancer)** | | | | | | | | |
|---|---|---|---|---|---|---|---|---|
| 6.Motor functions and mobility | 7.Exercise and fitness | 8.Activities of daily living | 9.Interpersonal interactions & relationships | 10.Education and vocation | 11.Community & social life | 12.Lifestyle modification | 13.Self-management | 14.Carer & family support |
| **0** | **0** | **0** | **0** | **0** | **0** | **0** | **0** | **0** |
| **0** | **0** | **0** | **0** | **0** | **X** | **0** | **X** | **0** |
| **X** | **0** | **0** | **0** | **0** | **0** | **0** | **0** | **0** |
| **X** | **X** | **0** | **0** | **0** | **0** | **0** | **0** | **0** |
| **X** | **0** | **0** | **0** | **0** | **0** | **0** | **X** | **X** |
| **0** | **0** | **X** | **0** | **0** | **0** | **0** | **0** | **0** |
| **0** | **X** | **0** | **0** | **0** | **0** | **0** | **0** | **0** |
| **0** | **X** | **0** | **0** | **0** | **0** | **0** | **0** | **0** |
| **0** | **0** | **0** | **0** | **0** | **0** | **0** | **X** | **0** |
| **X** | **0** | **X** | **0** | **0** | **0** | **0** | **0** | **0** |

| *Authors* *26-28* | *No of participants* | *Type of tumour* | *Rehabilitation intervention* | *Rehabilitation target 1-5 (WHO interventions for rehabilitation in cancer)* | | | | |
|---|---|---|---|---|---|---|---|---|
| | | | | 1. Mental/cognitive functions | 2.Pain management | 3.Bowl and bladder management | 4.Sexual functions & intimate relationships | 5.Cardiovascular and immunological functions |
| Van der Linden et al., 2021 | N=62 | Primary brain tumor patients - low-grade glioma & meningioma | Cognitive rehabilitation program (ReMind) tablet-based | **X** | **0** | **0** | **0** | **0** |
| Yoon et al., 2015 | N=40 | Patients with upper-extremity (UE) after brain tumour | Virtual reality-based rehabilitation | **0** | **0** | **0** | **0** | **0** |
| Zucchella et al., 2013 | N=58 | Inpatients with primary brain tumors | Early cognitive rehabilitation | **X** | **0** | **0** | **0** | **0** |

| **Rehabilitation target continued 6-14 (WHO interventions for rehabilitation in cancer)** | | | | | | | | |
|---|---|---|---|---|---|---|---|---|
| 6.Motor functions and mobility | 7.Exercise and fitness | 8.Activities of daily living | 9.Interpersonal interactions & relationships | 10.Education and vocation | 11.Community & social life | 12.Lifestyle modification | 13.Self-management | 14.Carer & family support |
| **0** | **0** | **0** | **0** | **0** | **0** | **0** | **X** | **0** |
| **X** | **0** | **X** | **0** | **0** | **0** | **0** | **0** | **0** |
| **0** | **0** | **0** | **0** | **0** | **0** | **0** | **0** | **0** |
